# Early combination therapy with etanercept and methotrexate in JIA patients shortens the time to reach an inactive disease state and remission: results of a double-blind placebo-controlled trial

**DOI:** 10.1186/s12969-020-00488-9

**Published:** 2021-01-06

**Authors:** Ekaterina Alexeeva, Gerd Horneff, Tatyana Dvoryakovskaya, Rina Denisova, Irina Nikishina, Elena Zholobova, Viktor Malievskiy, Galina Santalova, Elena Stadler, Larisa Balykova, Yuriy Spivakovskiy, Ivan Kriulin, Alina Alshevskaya, Andrey Moskalev

**Affiliations:** 1 Federal State Autonomous Institution, National Medical Research Center of Children’s Health, Moscow, Russian Federation; 2 Federal State Autonomous Educational Institution of Higher Education I.M. Sechenov First Moscow State Medical University of the Ministry of Health of the Russian Federation, Moscow, Russian Federation; 3 Asklepios Clinic Sankt Augustin, General Paediatrics, Arnold-Janssen-Straße 29, 53757 Sankt Augustin, Germany; 4 Department of Pediatric and Adolescent medicine, Medical Faculty, University Hospital of Cologne, Cologne, Germany; 5 V.A. Nasonova Research Institute of Rheumatology, Moscow, Russian Federation; 6 Federal State Educational Institution of Higher Education Bashkir State Medical University of the Ministry of Health of the Russian Federation, Ufa, Russian Federation; 7 State Samara Medical University, Samara, Russian Federation; 8 Medical Institute of National Research Ogarev Mordovia State University, Saransk, Russian Federation; 9 Saratov State Medical University n.a. V.I. Razumovsky, Saratov, Russian Federation; 10 Biostatistics and Clinical Trials Center, Novosibirsk, Russian Federation

**Keywords:** TNF inhibitor, Juvenile idiopathic arthritis, Remission

## Abstract

**Background:**

Remission is the primary objective of treating juvenile idiopathic arthritis (JIA). It is still debatable whether early intensive treatment is superior in terms of earlier achievement of remission. The aim of this study was to evaluate the effectiveness of early etanercept+methotrexate (ETA+MTX) combination therapy versus step-up MTX monotherapy with ETA added in refractory disease.

**Methods:**

A multi-centre, double-blind, randomized study in active polyarticular JIA patients treated with either ETA+MTX (*n* = 35) or placebo+MTX (*n* = 33) for up to 24 weeks, followed by a 24-week open-label phase. The efficacy endpoints included pedACR30 criteria improvement at week 12, inactive disease at week 24, and remission at week 48. Patients who failed to achieve the endpoints at week 12 or at week 24 escaped to open-label ETA+MTX. Safety was assessed at each visit.

**Results:**

By intention-to-treat analysis, more patients in the ETA+MTX group reached the pedACR30 response at week 12 (33 (94.3%)) than in the placebo+MTX group (20 (60.6%); *p* = 0.001). At week 24, comparable percentages of patients reached inactive disease (11 (31.4%) vs 11 (33.3%)). At week 48, 11 (31.4%) and eight (24.2%) patients achieved remission. The median (+/−IQR) times to achieve an inactive disease state in the ETA+MTX and placebo+MTX groups were 24 (14–32) and 32 (24–40) weeks, respectively. Forty-four (74/100 patient-years) adverse events (AEs) were reported, leading to treatment discontinuation in 6 patients.

**Conclusions:**

Early combination therapy with ETA+MTX proved to be highly effective compared to the standard step-up regimen. Compared to those treated with the standard regimen, more patients treated with a combination of ETA+MTX reached the pedACR30 response and achieved inactive disease and remission more rapidly.

## Background

Juvenile idiopathic arthritis (JIA) is the most common chronic rheumatic disease characterized by chronic arthritis with no further cause [1]. JIA is a diagnosis of exclusion and embraces a rather heterogeneous patient cohort [2]. Nevertheless, the most pronounced clinical and laboratory manifestations in these patients allow them to be combined into several JIA categories with respect to the International league of Associations for Rheumatology (ILAR) criteria [3]. For several JIA categories, own recommendations for patient management and treatment regimen exist [4]. Patients with polyarticular JIA who have no systemic manifestations can be treated with either non-steroidal anti-inflammatory drugs (NSAIDs) or intra-articular injections of glucocorticoids (GCs) as the first-line therapy depending on the presence of poor prognosis features. The efficacy of methotrexate (MTX) in polyarticular JIA has been demonstrated, and thus, it is recommended as the first disease modifying anti-rheumatic drug (DMARD) [4, 5]. However, not all patients respond sufficiently to MTX, and some are intolerant of its side effects [6, 7]. According to international guidelines and recommendations, JIA patients refractory to MTX treatment are eligible for treatment with biologics [4]. In most cases, biologics are prescribed after a patient has already been unsuccessfully treated with other drugs for several months. Approval of biologics such as Adalimumab, Etanercept, Golimumab and Tocilizumab is restricted to polyarticular patients who failed pretreatment with MTX. The current treatment strategies and the sequence of medication switching allow a considerable percentage of patients to achieve long-term remission [8]. However, most questions related to the optimal treatment regimen still need to be solved. Some of these questions have been outlined in the project plan for new ACR guidelines [9] that will be issued in 2021. In particular, the question regarding anti-tumour-necrosis –factor (anti-TNF) drugs (the first-line biologics for treating arthritis without systemic manifestations) consists of choosing between biologic monotherapy and biologic+non-biologic DMARD combination therapy if a biologic needs to be added to the treatment regimen. Identifying patient categories for the optimal treatment choice is also a high-priority task. Furthermore, according to the current clinical guidelines, anti-TNF agents can be prescribed only after the disease activity remains medium or high regardless of MTX treatment for 3–6 months [4]. Such a delay prolongs the time with active arthritis, reduces the current quality of life of patients and their parents and increases the risk of developing irreversible osteoarticular changes. Therefore, a very important issue is related to changing the timing of medication switching or identifying certain subgroups/cohorts of patients for whom early treatment with biologics or combination therapy will be the most effective option. Etanercept (ETA) remains one of the most frequently used anti-TNF drugs for JIA patients [10, 11]. The development and spread of biosimilars also contribute to their wider application [12, 13]. Choosing the optimal regimen of ETA therapy is very relevant for both issues mentioned above. Even for methotrexate, which is the basic therapy option, the relationship between the duration of the disease and the effectiveness of treatment has been demonstrated to be well known [14]. One of the key questions is whether the strategy of waiting for 3–6 months to determine whether MTX monotherapy is ineffective before prescribing ETA is beneficial compared to the simultaneous prescription of a combination treatment at baseline in terms of the time to achieve remission and the long-term outcome. Wallace et al. [15] attempted to demonstrate that early aggressive therapy contributes to the earlier onset of clinically inactive disease. However, in this study, the control group received MTX monotherapy, while the main group received combination MTX + ETA+GCs therapy. That combination made it impossible to assess the efficacy of the biologic itself. Therefore, we planned and conducted a double-blind, placebo-controlled study to evaluate the effectiveness of two different treatment regimens: ETA+MTX combination therapy vs the standard MTX monotherapy with ETA added subsequently (not earlier than after 12 weeks of MTX monotherapy) in JIA patients without systemic manifestations of disease.

## Methods

### Patient selection and overall study design

This multicentre, prospective, randomized, placebo-controlled study was conducted at seven paediatric rheumatology centres in the Russian Federation. Centralized randomization ensured that the patients were divided into two groups with a 1:1 allocation ratio using randomization envelopes. The study involved 3 phases. Phase 1 corresponded to 0–12 weeks (the double-blind phase); phase 2 to 12–24 weeks; and phase 3 to 24–48 weeks. The Initial Combination Scheme cohort was treated with ETA+MTX from baseline. The control therapy (Standard Consequent Scheme) group received placebo+MTX instead; unblinded treatment with ETA in both cohorts was performed as rescue therapy if the minimal clinical effectiveness criteria of paediatric American College of Rheumatology (PedACR)30 had not been reached at 12 weeks. At 24 weeks, patients were assessed for the presence of an inactive disease state according to the Wallace criteria, and those who had not reached the target also received rescue therapy. Patients without rescue remained unblinded until the end of the study. Final assessment was performed after 48 weeks. All patients were diagnosed with active polyarticular JIA as determined by the International League of Associations for Rheumatology (ILAR) criteria [3]; disease duration was at least 6 weeks. Active disease was defined as the presence of at least 4 active joints, a physician’s assessment of global disease severity of at least 3 of 10, and a patient’s or parent’s global assessment of wellbeing of at least 3 of 10 on a visual analogue scale (VAS). Female or male patients aged 2 to 17 years diagnosed with polyarticular JIA and disease durations of < 6 months were eligible. All patients had to have active JIA, i.e., > 3 joints with active arthritis, i.e., the presence of joint swelling or, in the absence of swelling, limitation of range of motion plus pain on motion and/or tenderness on palpation, and had to be naïve for treatment with biological drugs.

*The inclusion criteria were as follows:*

- Polyarticular JIA patients aged 2–17 years;
- Male or female patients;
- No current treatment with disease-modifying antirheumatic drugs (DMARDs)
- Therapy with other DMARDs (leflunomide, azathioprine, hydroxychloroquine, chloroquine, etc.) must have been stopped no later than 4 weeks prior to enrolment.

– Exception: the patient was allowed to receive stable doses of sulfasalazine provided that this treatment was received throughout the entire study, at baseline, and at least for four weeks prior to enrolment.

The patient was allowed to receive stable doses of NSAIDs and corticosteroids (≤ 0.2 mg/kg prednisolone per day, with the highest dose 10 mg/day) no later than 4 weeks before study initiation.

*The exclusion criteria were as follows:*

- Active joint count < 4;
- Physician’s assessment of disease severity: Visual analogue scale (VAS) score < 3 out of 10;
- Assessment of well-being by the patient or his/her parents: VAS score < 3 out of 10;
- Chronic or acute infection or severe infection episodes that required hospitalization or intravenous administration of antibiotics 30 days prior to study initiation;
- A previous history of malignancy;
- Pregnancy or lactation;
- Females who were unwilling to use proper methods of contraception or sexual abstinence;
- Ongoing active gastrointestinal disorders (e.g., inflammatory bowel disease);
- A previous history of tuberculosis or any opportunistic infection, including herpes zoster;
- A history of any chronic disease (except for JIA) that could influence the effectiveness or safety of the investigational medicinal product in investigator’s opinion.

### Treatment regimen

At treatment initiation, the Initial Combination Scheme cohort received ETA at a dose of 0.8 mg/kg per week (up to 50 mg/week) + MTX at a dose of 10–15 mg/week either orally or by subcutaneous injection as per the standard of the centre. The control cohort received placebo + MTX at a dose of 10–15 mg/week. If indicated, patients in both groups were also treated with NSAIDs, folic acid, and prednisolone at the investigator’s discretion.

### Outcome criteria

The primary outcome parameters of the study were improvement according to the pedACR30 criteria [16] at week 12. The PedACR core set parameters consist of (i) physician’s global assessment of disease activity (PhysVAS) on a 10 cm visual analogue scale (VAS); (ii) parent’s/patient’s global assessment of overall well-being (PatVAS) on a 10 cm VAS; (iii) the Childhood Health Assessment Questionnaire (CHAQ); (iv) the number of joints with active arthritis, defined by the presence of swelling or, if no swelling is present, limitation of motion accompanied by pain, tenderness or both; (v) the number of joints with a limited range of motion; and (vi) the erythrocyte sedimentation rate (ESR). The secondary outcome parameters included inactive disease according to the Wallace criteria [17] at week 24 and remission defined as continuous inactive disease for at least 24 weeks at week 48 [17]. The definition of ACR-inactive disease was according to Wallace et al. [11], requiring no active uveitis or arthritis; no fever, rash, splenomegaly, serositis, generalized lymphadenopathy or elevation of ESR/C-reactive protein (CRP); best possible PhysVAS; and duration of morning stiffness of ≤15 min.

### Phases and time points

Patients in the initial combination scheme group were treated with ETA+MTX since the first day of treatment. Patients receiving the standard consequent scheme were treated with placebo+MTX since the first day of treatment. At the week 12 visit, a primary assessment of improvement according to the pedACR30 criteria was performed. The responders continued to receive the initial blinded therapy. The non-responders were switched to the open-label phase and further received the ETA+MTX combination treatment. Secondary assessment of treatment effectiveness was performed at the week 24 visit based on whether the patients had reached an inactive disease state according to the Wallace criteria. Patients who had reached an inactive disease state continued to receive the earlier therapy. Patients who failed to reach an inactive disease state were switched to the open-label ETA+MTX combination treatment. Final effectiveness and safety assessments were performed at the week 48 visit. The patients were asked to make an additional follow-up study final visit 2–8 weeks after the end of the study. The intermediate points at which patients visited the study site and laboratory data were collected corresponded to 4, 8, 16, 42, and 40 weeks after study initiation.

### Ethical considerations

The study protocol was approved by the ethics committee of the Scientific Center of Children’s Health and was registered with the European Clinical Trials Database (EudraCT) as 2015–003384-11. The study was conducted according to the Good Clinical Practice standards. These standards ensured that the design, implementation, and communication of data were reliable; patients’ rights were protected; and the integrity of subjects was maintained by the confidentiality of their data. All patients and their parents provided written informed consent in accordance with the Declaration of Helsinki, which included their consent for using their data in analyses and to be presented.

### Protocol for collecting the effectiveness and safety data

Clinical and laboratory values were monitored at each visit for each patient. The parameters of disease activity and severity were evaluated, including: evaluation of the joints (the swollen joint count, tender joint count, the number of joints with limitation of motion (LOM), physician’s global assessment of disease activity, Patient’s global assessment of wellbeing, the CHAQ (Childhood Health Assessment Questionnaire) score [18], the Juvenile Arthritis Disease Activity Score (JADAS 71) [19], inactive disease state according to the Wallace criteria [17], and the pedACR 30/50/70/90/100 response [3]. The primary objective of the study was to compare the effectiveness of the combination treatment with etanercept/methotrexate to the standard consequent scheme of treatment with methotrexate (MTX) according to the number of responders who reached an inactive disease state/remission at weeks 24 and 48 and the time required to reach these parameters. The secondary objectives of the study consisted of evaluating and comparing the effectiveness of MTX monotherapy and ETA+MTX combination therapy using the pedACR 30/50/70/90/100 criteria and evaluating the safety of etanercept in JIA patients. All AEs were reported to be in compliance with the Common Terminology Criteria for Adverse Events and classified according to the Medical Dictionary for Regulatory Activities (MedDRA).

### Statistical analysis

The calculations were performed using the R Statistical Package (http://www.r-project.org). Descriptive statistics are shown as absolute frequencies or medians with interquartile ranges. The Mann-Whitney U-test, ANOVA, Pearson’s χ^2^ test, or non-parametric Kruskal–Wallis test by rank and median multiple comparisons were used depending on the type of the analysed data. All the reported *p*-values were based on two-tailed tests of significance; p-values < 0.05 were regarded statistically significant. STATISTICA 7.0 software (StatSoft, USA) and RStudio software version 1.0.136 (Free Software Foundation, Inc., USA) with R package version 3.3.1 (R Foundation for Statistical Computing, Austria) were used for the analyses.

## Results

### Baseline characteristics

**Fig. 1:**
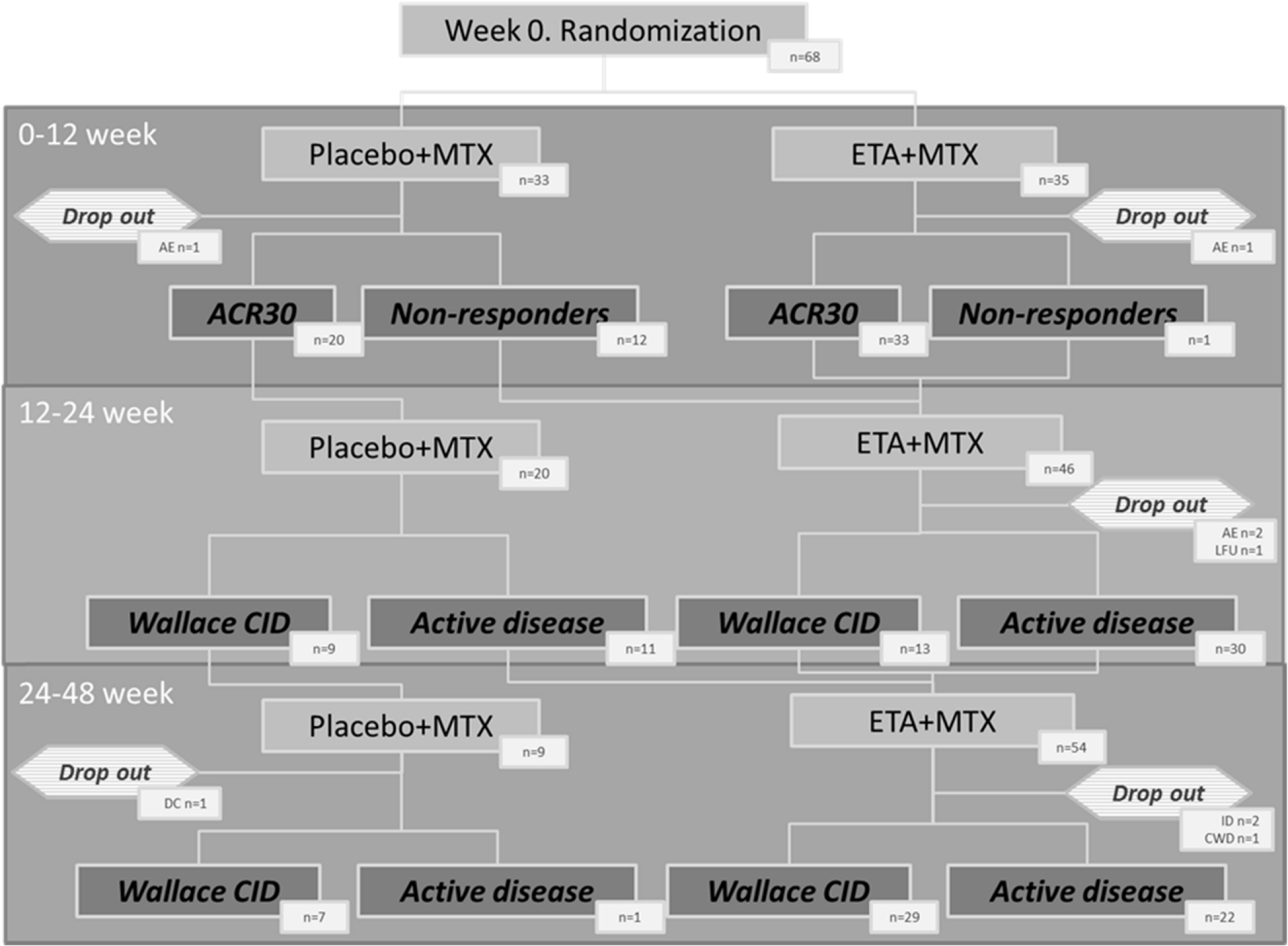
Study scheme with the patient flow chart. AE = adverse events, LFU = lost to follow-up, CWD = consent withdrawal, DC = diagnosis changed, CID = clinical inactive disease; ID = investigator decision; ETA = Etanercept, MTX = methotrexate

The study involved 68 patients: 35 patients were randomized in the cohort receiving ETA+MTX combination therapy and 33 patients were randomized to be treated with placebo+MTX. Table 1 summarizes the data of the patients’ characteristics at baseline and arthritis severity and activity at the initiation of the current treatment. The patients were comparable in terms of sex, age, distribution of JIA categories and prior treatment. However, several parameters differed between the cohorts. Patients in the ETA-MTX combination cohort were older at disease onset and at diagnosis and had higher levels of CRP, JADAS-71 and physician’s assessment of disease activity. Figure 1 outlines the patient disposition with information about patient switching and the main outcomes during the study. In total, 68 patients were enrolled and randomized: 33 received placebo+MTX and 35 received ETA+MTX (Fig. 1); of these, 2 patients discontinued the study before week 12, leaving 32 patients in the placebo+MTX group and 34 in the ETA+MTX eligible for analysis at week 12. Twelve pedACR30 nonresponders in the placebo+MTX group and 1 nonresponder in the ETA+MTX group received open-label ETA+MTX after week 12. Between week 12 and week 24, there were 3 additional drop-outs, leaving 20 patients in the placebo+MTX group and 43 in the ETA+MTX group eligible for analysis at week 24. From the 63 patients entering part 2, only 4 discontinued the protocol between week 24 and week 48.

**Table 1:** Baseline demographic, anamnestiс, clinical and laboratory characteristics of JIA severity in patients enrolled in the study

Parameter	ETA + MTX (***n*** = 35)	Placebo + MTX (***n*** = 33)	p
*Demographic characteristics*			
Sex:			0.471
female, n (%)	22 (64.71%)	25 (75.76%)	
male, n (%)	12 (35.29%)	8 (24.24%)	
Age at enrolment, years; median (IQR range)	9.8 (5.82–12.94)	6.62 (4.1–13.26)	0.11
Disease duration (starting from the onset symptoms) to baseline years; median (IQR range)	0.81 (0.29–1.68)	0.74 (0.32–3.48)	0.632
Age of onset of specific complaints years; median (IQR range)	7.2 (4.38–10.82)	4.7 (2.4–9.44)	**0.032**
Age at diagnosis years; median (IQR range)	9.16 (5.4–12.05)	4.86 (2.49–11.37)	**0.035**
Disease duration until diagnosis, years	2.5 (1–9)	3 (2–16)	0.38
JIA category			
Seronegative Polyarthritis, n (%)	21 (60%)	16 (48%)	0.47
Seropositive Polyarthritis, n (%)	1 (2.9%)	0	1.0
Extended Oligoarthritis, n (%)	8 (23%)	12 (36%)	0.29
Polyarticular enthesitis associated arthritis, n (%)	5 (14.3%)	3 (9%)	0.71
Unclassified polyarticular JIA, n (%)	0	2 (6%)	0.23
*Prior treatment*			
Conventional NSAIDs, n (%)	31 (88.57%)	27 (81.82%)	0.507
COX-2 inhibitors, n (%)	11 (32.35%)	13 (39.39%)	0.729
Sulfasalazine, n (%)	6 (17.14%)	9 (27.27%)	0.475
Leflunomide, n (%)	1 (2.86%)	0 (0%)	> 0.999
Oral GCs, n (%)	1 (2.86%)	2 (6.06%)	0.608
Other GCs, n (%)	11 (31.43%)	8 (24.24%)	0.697
*Clinical and laboratory characteristics*			
Swollen joint count, median (IQR range)	6 (4–9)	4 (2–7)	0.018
Tender joint count, median (IQR range)	6 (4.5–12.5)	6 (4–12)	0.349
Number of joints with LOM, median (IQR range)	6 (4.5–10.5)	6 (3–14)	0.521
Number of active joints, median (IQR range)	7 (6–11.5)	7 (4–12)	0.191
Haemoglobin, median (IQR range)	12 (11.45–12.8)	12 (11.4–12.5)	0.726
ESR, median (IQR range)	12 (2–20)	12 (8–23)	0.301
CRP, median (IQR range)	3.5 (1–13.45)	0.7 (0–9)	0.003
JADAS-71, median (IQR range)	24 (18–34)	19 (17–24.3)	0.025
CHAQ, median (IQR range)	1.5 (0.75–1.75)	1 (0.5–1.5)	0.057
Physician’s assessment of disease activity using VAS, median (IQR range)	8 (6–9)	6 (5–7)	0.014
Patient’s assessment of well-being using VAS	8 (5–9)	7 (5–8)	0.114
Patient’s assessment of disease activity using VAS, median (IQR range)	7.5 (5–8)	7 (5–8)	0.353

*P* values are based on the Mann-Whitney U-test (for continuous data) and on Pearson’s χ2 test (for categorical data)

### PedACR response rates during the first 12 weeks of ETA+MTX combination therapy vs placebo+MTX

**Fig. 2:**
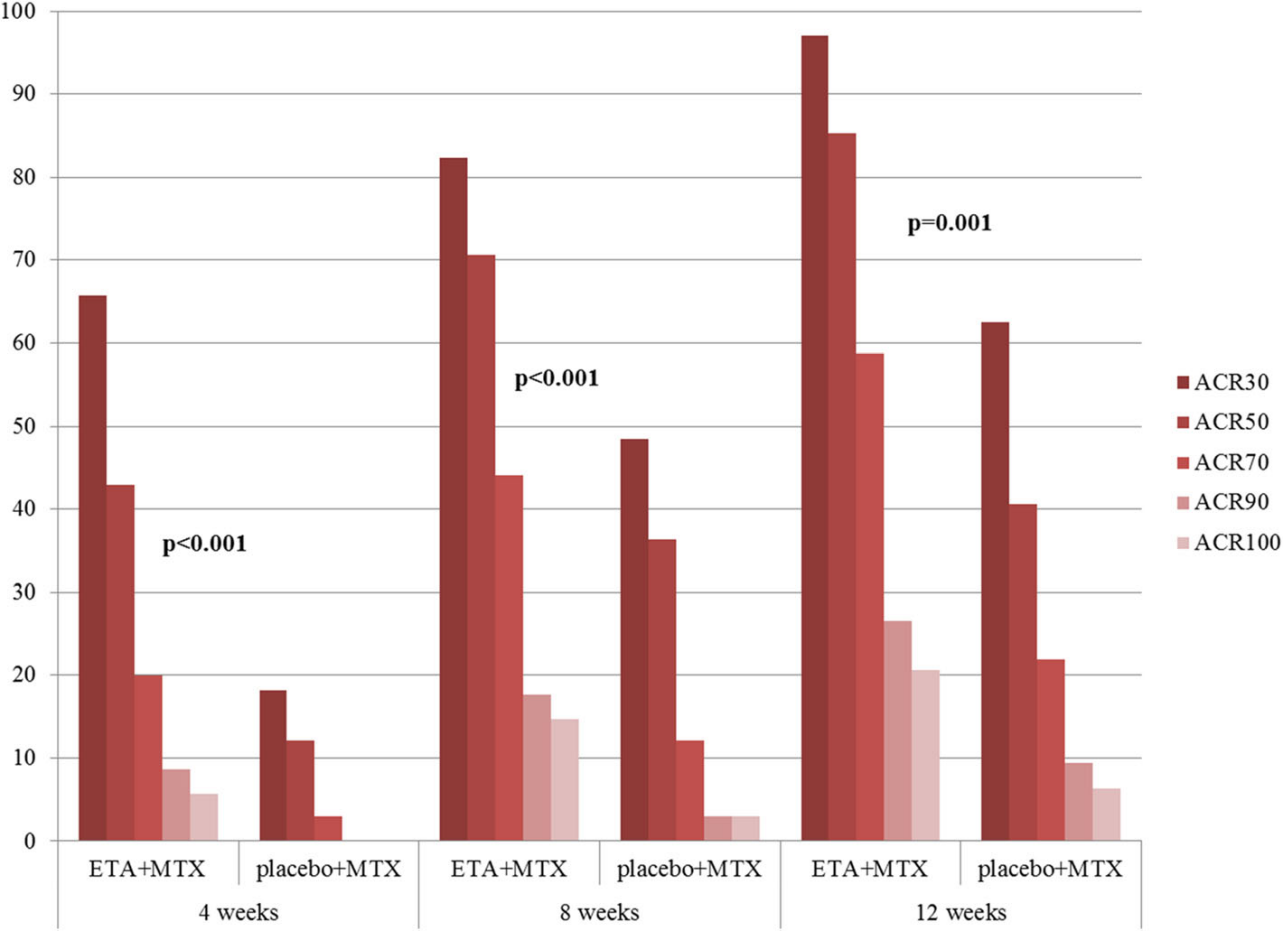
Dynamics of patients’ condition in the study groups evaluated using the ACR Pedi criteria. ETA = Etanercept; MTX = Methotrexate. *P* values are based on Pearson’s χ2 test (for categorical data)

**Fig. 3:**
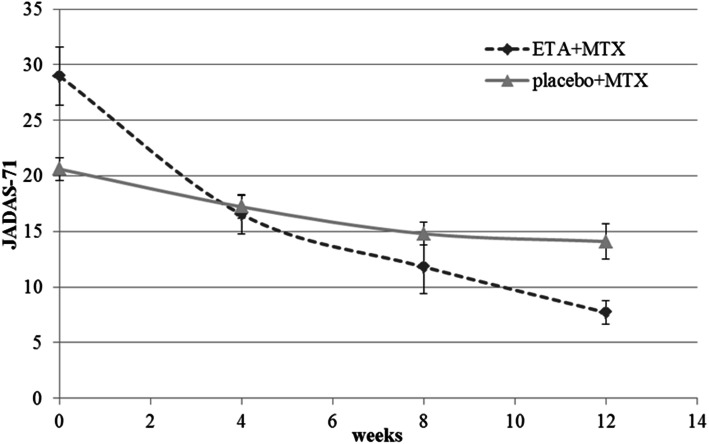
Dynamics of JADAS-71 in the treatment groups during the first 12 weeks of therapy. ETA = Etanercept; MTX = Methotrexate. Whiskers show standard errors of the mean

The scale and kinetics of the response according to the pedACR30/50/70/90/100 criteria and the JADAS-71 scores during the first 12 weeks of treatment are shown in Figs. 2–3. Differences between the cohorts were highly significant at all points of time, favouring the early combination treatment.

JADAS-71 significantly decreased in both groups during the first 12 weeks of treatment: the median level reduced from 19 (17–24.3) at baseline to 12 (9–19) at week 12 in the placebo+MTX group and from 24 (18.5–34) to 6 (3.25–9.75) in the ETA+MTX group. The dynamics of the reduction differed between the study groups: the decrease in JADAS-71 was more rapid and significant in the group treated with ETA+MTX. The reduction of JADAS-71 was 18.3 (12–26.2) in the ETA+MTX group, which was significantly higher than 6 (2.5–10) in the placebo+MTX group (*p* < 0.001). We additionally compared placebo+MTX responders with ETA+MTX responders. Among the patients treated with placebo+MTX, the subgroups of responders and nonresponders were also compared. In the group initially treated with ETA+MTX, 94% of patients responded to therapy within the first 12 weeks by reaching pedACR30. Meanwhile, only 60% of patients with mild JIA in the cohort responded to placebo+MTX therapy. The responders to placebo+MTX had a significantly milder course of the disease in terms of tender joint count, number of joints with limitation of motion, and VAS and JADAS-71 disease activity scores than both ETA+MTX responders and MTX non-responders (Table 2). Table 3 summarizes the patients who reached the clinical criteria of treatment effectiveness in this study.

**Table 2:** Comparison of the baseline parameters in the subgroups of ACR30 responders and non-responders after 12 weeks of treatment

	ETA + MTX, week 12 ACR30 responders (***n*** = 33)	Placebo + MTX, week 12 ACR30 responders (***n*** = 20)	Placebo + MTX, week 12 ACR30 non-responders (***n*** = 12)	p
Number of joints with LOM	8 (5–17)	6 (4–7.5)	5 (3.5–10.5)	**0.030**
Tender joint count	10 (5–19)	6 (4–8)	6 (3.5–10.5)	**0.023**
VAS disease Activity (physician’s)	8 (6–9)	6 (6–7)	6.5 (4.5–8)	**0.036**
JADAS-71	24 (19–34)	19.3 (19–23.8)	17 (14–27)	**0.020**
CRP level	3.6 (1–26)	1.6 (0.7–9.4)	1.1 (0.1–2.9)	**0.031**

Comparison of the baseline parameters of the different treatment regimens: the standard scheme including the consequent addition of ETA to non-responders to MTX monotherapy vs the scheme with ETA + MTX combination therapy received over 12 weeks

*P* values are based on the non-parametric Kruskal–Wallis test by rank and median multiple comparisons

**Table 3:** Intention-to-treat and per protocol analysis of patients according to the ACR30 response after 12 weeks of treatment and reaching the Wallace inactive disease criteria after treatment for 24 and 48 weeks on the basis of the initial patient allocation into groups

Time-point	Efficacy parameter achieved	Intention-to-treat			Per protocol		
		Initial combination scheme ETA + MTX	Standard consequent scheme with MTX^**a**^	p	Initial combination scheme ETA + MTX	Standard consequent scheme with MTX^a^	p
**12**	**ACR30**	33/35 (94.3%)	20/33 (60.6%)	**0.001**	33/34 (97.1%)	20/32 (62.5%)	**0.001**
**24**	**Wallace inactive disease**	11/35 (31.4%)	11/33 (33.3%)	> 0.999	11/33 (33.3%)	11/30 (36.7%)	0.798
**48**	**Wallace inactive disease**	17/35 (48.6%)	20/33 (60.6%)	0.342	17/32 (53.1%)	19/29 (65.5%)	0.436
**48**	**Remission**	11/35 (31.4%)	8/33 (24.2%)	0.594	11/32 (34.4%)	8/29 (27.6%)	0.593

^**a**^According to treatment regimen in this group, non-responders were supposed to switch to open-label ETA+MTX combination therapy after 12 or 24 weeks of treatment if they failed to reach the effectiveness parameter corresponding to the given week

*P* values are based on Pearson’s χ2 test (for categorical data)

### Adherence and reasons for discontinuation

Two patients were withdrawn from the study because of AEs during the first 12 weeks of treatment. One patient in the ETA+MTX group had an injection site reaction (a*rthralgia, myalgia, bone pain after injections*), and one patient in the placebo+MTX group developed hepatotoxicity after 8 weeks of treatment. In the intention-to-treat analysis, these patients were regarded as non-responders after 12 weeks of treatment (according to the ACR30/Wallace criteria, respectively) and were excluded from the per protocol analysis. Three patients were withdrawn from the study between week 12 and week 24 of treatment. One of these patients was from the group receiving combination therapy since study initiation. This patient was lost to follow-up: the patient’s data were taken into account in the intention-to-treat analysis but were not taken into account for the time points of 24 and 48 weeks in the per protocol analysis. Two patients who initially received placebo+MTX and were switched to open-label treatment after 12 weeks were withdrawn because of AEs, one thrombocytopenia and one with hepatotoxicity. These patients were regarded as non-responders in both analyses. Four patients were withdrawn from the study between week 24 and week 48 of treatment. In the group receiving combination treatment since study initiation, one patient withdrew his/her consent. The patient was not taken into account at the time point “48 weeks” in the per protocol analysis. Two patients were withdrawn according to the investigator’s decision due to poor efficacy, which was regarded as secondary ineffectiveness. One patient receiving placebo+MTX was withdrawn because his diagnosis was changed to juvenile dermatomyositis. The patient was not taken into account for the time point “48 weeks” in the per protocol analysis. The median time to reach an inactive disease state according to the Wallace criteria differed for the treatment groups: 24 (14–32) weeks in the ETA+MTX group and 32 (24–40) weeks in the placebo+MTX group. Time to achieve remission also differed between the groups. In all eight patients who achieved remission in the standard consequent scheme with MTX, this time was 48 weeks. Meanwhile, in 5 of 11 patients who achieved remission in the initial combination regimen ETA+MTX, this time was less than 48 weeks (after 32 weeks of treatment in 2 patients; after 36 weeks of treatment in 2 patients, and after 42 weeks of treatment in 1 patient). Thus, until the end of the study, patients had active arthritis for 32 (24–52) weeks and 6 (5–9) visits in the ETA+MTX group and for 40 (24–50) weeks and 7 (5–9) visits in the placebo+MTX group.

### Safety analysis

Treatment safety was evaluated separately for each phase, with allowance for the therapy received by the patients (Table 4). At all phases, the frequencies of patients experiencing AEs in the ETA+MTX group were 11% (6 AEs), 11% (5 AEs), and 17% (14 AEs) and in the MTX + PLC cohort were 21% (11AEs), 15% (3 AES), and 33% (3 AEs) during phases 1, 2, and 3, respectively. Serious adverse events were reported only for the MTX group during phase 3 (2 SAEs): (1) uveitis and (2) patient’s diagnosis was changed to a different autoimmune disease. AEs that were not serious but still required additional actions and therapy included infections, gastrointestinal diseases and hepatic events. Infections were the most frequent AE (50%/40%/57% in the ETA+MTX group and 36%/100%/33% in the MTX group during phases 1, 2, and 3). Altogether, there were six infectious events treated with antibiotics. There were four patients from the ETA/MTX cohort: Phase 1: Acute respiratory infection *n* = 1; Phase 3: Acute bronchitis *n* = 2; acute respiratory infection *n* = 1. There were 2 patients from the MTX + PLC cohort: Phase 1: Fever+cough+rhinitis n = 1; phase 3: Acute respiratory infection n = 1. The frequency of AEs classified as gastrointestinal diseases and hepatic events was rather high at initiation of MTX monotherapy (a total of 54% during phase 1 and 0% during phases 2 and 3). An opposite situation was observed in the ETA+MTX group, for which these adverse events were reported after 12 weeks of treatment (phases 2 and 3) rather than at treatment initiation.

**Table 4:** Number of patients with adverse events in the ETA+MTX and MTX groups according to study phase

Phase	Group	
	MTX	ETA + MTX
**Phase 1**	7 patients (21%) (11 AEs)	4 patients (11%) (6 AEs)
**Phase 2**	3 patients (15%) (3 AEs)	5 patients (11%) (5 AEs)
**Phase 3**	3 patients (33%) (3 AEs)	9 patients (17%) (14 AEs)

The table shows the number of patients with AE, (rate of patients with AE), (number of AE); *AE* adverse event, *ETA* Etanercept, *MTX* Methotrexate

## Discussion

Our study revealed that initial therapy with ETA+MTX was already very effective after weeks 4 to 12 of treatment, with nearly all patients responding according to the pedACR30 criteria, and the response rate was significantly higher than in the placebo+MTX group. Although ETA has been studied in polyarticular JIA for 20 years, this is the first head-to-head placebo-controlled trial with ETA in these patients. A different design was used in the present study than in the TREAT study, in which patients received ETA+MTX + high-dose steroids or MTX plus two placebos for steroids and ETA [15].

The primary objective of our study was to investigate whether ЕТА + МТХ combination therapy is superior than the treatment regimen recommended by current clinical guidelines (MTX only with the subsequent addition of ETA if necessary). We have demonstrated that both treatment regimens are equally effective for achieving remission at week 48. Approximately 50% of patients reached an inactive disease state after 48 weeks of treatment. However, the effectiveness of these treatment regimens differed in terms of the time required to reach an inactive disease state. It took a median of 24 (IQR 14–32) weeks to reach an inactive disease state for patients treated with the ETA+MTX combination. Patients who had been initially treated with placebo+MTX and then switched to combination therapy (at any time) reached an inactive disease state after a median of 32 (IQR 24–40) weeks. The time to reach an inactive disease state significantly affects quality of life of patients and their parents.

The clinical response to methotrexate is known to have a delayed onset. It is thought that the polyglutamated form of methotrexate is responsible for its DMARD activity. There is usually a time lag in the efficacy of low-dose methotrexate in clinical practice, as accumulation of intracellular polyglutamated methotrexate is a slow process [20], which might explain this delayed response to MTX monotherapy. TNF inhibitors, however, often show an immediate onset of clinical improvement in the first weeks [21].

Furthermore, the investigators believe that reaching an inactive disease state in the early stage of arthritis (up to 2 years after the onset) may prevent the development of irreversible osteoarticular changes and reduce the future risk of disability since the earlier the treatment with biological preparations is started in an aggressive course of the disease, the more chances for the reversibility of pathological changes. In fact, the delay in identifying the optimal treatment at an early stage of disease can influence long-term joint damage [22].

When preparing novel clinical guidelines, experts should take into account a number of questions related to the identification of the optimal administration of biologics (monotherapy versus combination with non-biologic DMARD). The subsequent questions should address the optimal use of each biologic, taking into account that there might be cases for whom biologic monotherapy is acceptable due to adequate patient response, adverse events, or other aspects. In particular, the question ‘should ETA monotherapy versus ETA + non-biologic DMARD be recommended for patients with polyarticular JIA?’ is still open. Similar questions are also open for all anti-TNF drugs. Furthermore, a double-blind placebo-controlled study to compare the effectiveness of MTX vs ADA + MTX therapy has been published [23]. Ramanan et al. demonstrated that adalimumab therapy controlled inflammation and was associated with a lower rate of treatment failure than placebo among children and adolescents with active JIA-associated uveitis who were taking a stable dose of methotrexate. Patients receiving adalimumab had a much higher incidence of adverse events and serious adverse events than those who received placebo. In another recent study comparing different treatment regimens [24], it was shown that in the long-term treatment strategy, there was no significant difference in the rate of achieving inactive disease. Nevertheless, the authors show that ACR30 has suitabledynamics for a large proportion of children on combination therapy with etanercept at the beginning of treatment. In this study, the difference with methotrexate monotherapy at 3 months is approximately 20%, which is consistent with our results [24].

MTX is recommended as the first-line treatment in oligoarthritis persisting despite nonsteroidal anti-inflammatory drugs (NSAIDs) and intraarticular steroid therapy and in all patients with active polyarticular disease [4]. However, the question regarding shortening of the period before switching to biologics from MTX monotherapy to shorten the time to reach an inactive disease state and improve patient’s quality of life is still open. Although MTX is the first-choice drug in JIA, approximately 50% of patients fail to respond to it, and even in responders, the grade of remission is low [25, 26]. Given the time lag between MTX treatment initiation and the patient response (approximately 3 months), it would be particularly useful to determine a priori the probability of a beneficial therapeutic response [22]. According to the results of this study, ETA+MTX combination therapy allows patients to achieve remission sooner than MTX monotherapy. This fact makes it necessary to revise the timing of biologics treatment initiation in the current clinical guidelines.

Potential responders and non-responders to MTX should also be identified for optimizing the treatment regimens. The first attempts to identify predictors of methotrexate response were made a rather long time ago. In 2010, Vilca et al. analysed 563 patients from the PRINTO database [27]. All patients received MTX monotherapy for 6 months. The authors demonstrated that the most important predictors of non-response were as follows: disease duration > 1.3 years, ANA negativity, higher CHAQ disability index, and presence of right and left wrist activity. Hence, children with a severe disease course and long disease duration exhibit the worst response, even to long-term MTX monotherapy. In this study, we have confirmed these findings and additionally demonstrated that 94% of patients treated with ETA+MTX since treatment initiation responded to ETA treatment and achieved ACR30 during the first 12 weeks. However, only 60% of patients responded to the placebo+MTX treatment in the respective group, while the disease course was significantly milder in these patients according to joints with a limited range of motion, tender joints, the VAS score, and JADAS. Hence, MTX quickly provides relief only to children with a mild course of JIA, while ETA+MTX can help all children, regardless of disease severity. Data from the German BIKER registry from an even larger patient population of 731 JIA polyarticular JIA patients treated with MTX showed that a minimal response of a pedACR30 was reached by 77.4% at month 3 and by 83.1% of patients at month 12, while 43.1 and 65.9% of patients had a PedACR 70 response at months 3 and 12, respectively [28]. Thus, a minimal response was frequently already reached at month 3, while a stronger response to MTX treatment took usually longer to achieve. In multivariate analysis, the determinants for reaching PedACR 70 at month 12 were a disease duration of less than 1 year, a lower number of tender but a higher number of active joints and the presence of morning stiffness at baseline. Importantly, in the study of Albarouni et al. patients reaching a pedACR30 response at month 3 have a 4-fold increased and, thus, a significantly higher chance to reach pedACR70 at month 12 [28]. Patients who do not have a pedACR30 response at month 3, therefore, should not continue to receive the same treatment. In this study, we also collected safety data for MTX treatment vs anti-TNF + MTX. During the first 12 weeks of combination therapy, adverse events were reported in 17% of patients, while 33% of patients treated with placebo+MTX developed adverse events. Infectious adverse events were the most frequent AEs in both treatment groups.

Our study has a number of limitations. The groups being compared differed significantly at baseline in terms of a number of parameters, including disease duration and disease severity according to the CHAQ score. Despite randomization, patients in the ETA+MTX group had a higher age of JIA debut than patients in the Placebo+MTX group of initially combined therapy. This difference may be due to the multicentre character of the study since the 6 centres participating in the study were geographically widely scattered over the territory of the Russian Federation. The differences may be due to some population and ethno-genetic characteristics of JIA in children in different regions. JIA does not represent a single disease but is a group of very heterogeneous categories. Thus, greater differences than in adult studies including rheumatoid arthritis patients can be expected. Moreover, JIA compared to RA is a very rare disease, and only much smaller study samples can be studied. A younger age at disease onset may have influenced the response rate in the Placebo+MTX group. However, despite this possible advantage, the level of response in the first 12 weeks in the Placebo+MTX group was significantly lower. All other differences (higher swollen joint count, higher CPR level and JADAS-71 level) were also disadvantages for the primary combination group, which did not prevent ETA+MTX patients from demonstrating a higher level of response. Nevertheless, we performed a unique comparison of two different treatment schemes in a multicentre Russian prospective trial that can be compared to data for patients from other countries.

## Conclusion

Our findings demonstrate that ETA+MTX combination therapy is a universal treatment regimen that can be used for patients with both mild and severe disease courses to quickly reduce inflammation and articular symptoms during the first 12 weeks of treatment. Only patients with a mild disease course respond quickly to MTX monotherapy. Patients treated with the ETA+MTX combination since treatment initiation reached an inactive disease state and remission more rapidly than patients for whom ETA was added only after they had failed to respond to MTX monotherapy. Hence, earlier addition of ETA to the treatment regimen shortens the time to achieve remission and improves patients’ quality of life. Both cohorts showed an acceptable safety profile.

## Data Availability

Data sharing is not applicable to this article, as no datasets were generated or analysed during the current study.

## Abbreviations

AE: Adverse event
CHAQ: Childhood Health Assessment Questionnaire
CRP: C-reactive protein
DMARD: Disease-modifying antirheumatic drug
ESR: Erythrocyte sedimentation rate
ETA: Etanercept
GCs: Glucocorticoids
ILAR: International League of Associations for Rheumatology
IQR: Interquartile range
JADAS: Juvenile Arthritis Disease Activity Score
JIA: Juvenile idiopathic arthritis
LOM: Limitation of motion
MTX: Methotrexate
NSAIDs: Nonsteroidal anti-inflammatory drugs
pedACR: American College of Rheumatology paediatric <criteria>
RF: Rheumatoid factor
TNF: Tumour necrosis factor
VAS: Visual analogue scale
